# Dietary manipulations for health and the prevention and management of disease: An editorial

**DOI:** 10.1113/EP092517

**Published:** 2025-05-05

**Authors:** Kelly A. Bowden Davies, Colleen S. Deane

**Affiliations:** ^1^ Department of Sport and Exercise Sciences, Institute of Sport Manchester Metropolitan University Manchester UK; ^2^ Human Development & Health, Faculty of Medicine University of Southampton, Southampton General Hospital Southampton UK

## 1 INTRODUCTION

This special issue of *Experimental Physiology* features a collection of papers stemming from the ‘Dietary Manipulations for Health and the Prevention and Management of Disease’ symposium of The Physiological Society, held at Manchester Metropolitan University in March 2024. The 2‐day programme brought together speakers from Australia, Canada, Denmark, the Netherlands and across the UK, spanning all career stages from postgraduate researchers to world‐leading experts who presented their latest findings and/or emerging methodologies. The small, focused meeting (∼60 delegates) fostered a dynamic environment that encouraged high‐quality scientific discussions and meaningful engagement. In this Editorial, we reflect on the origins of the symposium and highlight how each article in this special issue contributes to advancing our understanding of the role of diet in health and disease.

### 1.1 Symposium origins

The symposium aimed to explore dietary strategies for enhancing health and preventing or managing disease, with a focus on identifying gaps in current knowledge and limitations in their application through the examination of original data. Our discussions covered a wide range of evidence, from mechanistic insights of inter‐organ signalling of adipose tissue to the effects of macronutrient composition, such as the role of protein intake in muscle protein synthesis and the impact of dietary fat on liver metabolism. To complement these discussions, we hosted a series of methodological workshops and symposium sessions, showcasing the latest techniques and technologies. These sessions bridged the gap between controlled laboratory settings and real‐world environments, featuring key methodologies such as omics approaches, magnetic resonance imaging and continuous glucose monitoring. The symposium also explored the benefits of traditional dietary interventions, including low‐calorie diets and time‐restricted eating, while considering novel approaches for disease prevention and management such as the food matrix and meal regularity. We are excited to share these articles that span organs, methods and applications from experimental physiology through to clinical application.

## 2 THE ROLE OF ADIPOSE‐DERIVED MOLECULES

Malicka et al. (2025) discuss inter‐organ signalling mechanisms of metabokines and lipokines derived from brown and beige adipose tissue and focus on their roles in metabolic homeostasis and diseases. This thorough review of the literature explores the clinical potential of therapeutically targeting metabokine‐mediated pathways, for example, to treat cardiometabolic disease. The authors propose that specific modulation of metabokine transporters and receptors may offer superior specificity and efficacy compared with traditional, and as of yet unsuccessful, therapeutic approaches to brown adipose tissue thermogenic activation.

## 3 PHENOTYPIC AND DIETARY INFLUENCES ON INTRAHEPATIC TRIGLYCERIDE ACCUMULATION

Smith et al. (2025) delve into the mechanisms governing intrahepatic triglyceride (IHTG) accumulation, emphasising the interplay between individual phenotypes and dietary factors. They explore how variables such as adiposity levels, insulin sensitivity and genetic predispositions can influence hepatic lipid metabolism, ultimately affecting the likelihood of IHTG deposition. The authors present evidence that high‐carbohydrate diets, particularly those rich in fructose, can exacerbate hepatic lipogenesis, while high‐fat diets contribute to IHTG accumulation through different metabolic pathways. This work enhances our understanding of non‐alcoholic fatty liver disease and paves the way for personalised dietary interventions aimed at reducing IHTG and its metabolic effects. This nuanced examination highlights the complexity of dietary impacts on liver fat content.

## 4 DIETARY PROTEIN AND MUSCLE PROTEIN SYNTHESIS ACROSS THE LIFESPAN

Brook (2025) explores our current knowledge on how dietary protein intake impacts muscle protein synthesis across age, and specifically details how the stable isotope deuterium oxide has permitted novel insights into free‐living assessments of muscle protein metabolism. Following a detailed review of the literature, Brook (2025) concludes that increasing protein quality, quantity and the leucine content can enhance long‐term muscle protein synthesis. In the context of ageing, evidence suggests that a protein intake of >1.2 g/kg/day is generally effective for increasing muscle protein synthesis in older adults, but anything below this would benefit from leucine supplementation and a higher proportion of higher‐quality proteins.

## 5 TIMING OF FASTED EXERCISE AND METABOLIC PHYSIOLOGY

McIver et al. (2025) explore the effect of fasted exercise timing (morning vs. evening) on metabolic, gastrointestinal and appetite responses in overweight males. Their data shows that both morning and evening fasted exercise improve glucose‐insulin dynamics without impacting gastric emptying, appetite or energy intake. This suggests that the metabolic benefits of fasted exercise are not time‐dependent, at least not in the acute context. The study highlights the potential role of fasted exercise in weight management strategies. It also lays the groundwork for future research into chronic adaptations and circadian interactions in metabolic regulation.

## 6 PHYSIOLOGICAL RHYTHMS AND SKELETAL MUSCLE METABOLISM

Betts et al. (2025) review emerging evidence from molecular, physiological and whole‐body studies to demonstrate how muscle‐specific circadian rhythms are influenced by feeding patterns, physical activity and metabolic cues. They also highlight how circadian misalignment, such as that caused by shift work or irregular lifestyle patterns, can disrupt these rhythms, potentially leading to insulin resistance and metabolic inflexibility. This review refocuses attention on the muscle as a key peripheral clock, offering important insights into the pathogenesis of metabolic diseases and the design of time‐sensitive interventions. By bridging molecular chronobiology with applied physiology, the authors set a clear direction for future research into how circadian alignment of behaviour and metabolism could optimise skeletal muscle health and overall metabolic outcomes.

## 7 THE TWIN CYCLE HYPOTHESIS: CONCEPT TO NHS IMPLEMENTATION

Taylor (2025) describes the latest update on the Twin Cycle Hypothesis, which suggests that chronic caloric excess triggers a self‐reinforcing cycle of hepatic and pancreatic fat accumulation, leading to insulin resistance and β‐cell dysfunction, explaining the pathogenesis of type 2 diabetes. Taylor's discussion goes beyond theoretical concepts, offering an empirical journey from hypothesis development to clinical validation. A key highlight is the DiRECT (Diabetes Remission Clinical Trial), which demonstrated that calorie‐restricted diets could reverse type 2 diabetes by depleting ectopic fat stores and restoring normoglycaemia. This evidence helped shape the national NHS programme in the UK, which provides structured weight management interventions aimed at achieving remission. This work exemplifies the power of mechanistic research to develop evidence‐based therapeutic approaches and shape public health strategies and clinical paradigms with significant societal impact.

## 8 SUMMARY

Collectively, the ‘Dietary Manipulations for Health and the Prevention and Management of Disease’ symposium and accompanying special issue emphasise the critical role diet and nutrition plays in health and disease. This special issue demonstrates the breadth of this topic, covering many different organs systems from muscle to adipose to liver tissue, and application of highly specialised and advanced methodologies such as the use of stable isotope tracers. Future research should focus on personalised approaches to nutrition, and on technologies and methodologies to enable this. Considering the rapid pace of the field, we propose that a biennial ‘Dietary Manipulations for Health and the Prevention and Management of Disease’ symposium and accompanying special issue would provide a fantastic platform and resource for academics and other key stakeholder's to stay ahead of the curve, whilst also facilitating multi‐disciplinary collaborations to tackle the biggest dietary healthcare challenges.

## AUTHOR CONTRIBUTIONS

Both authors have read and approved the final version of this manuscript and agree to be accountable for all aspects of the work in ensuring that questions related to the accuracy or integrity of any part of the work are appropriately investigated and resolved. All persons designated as authors qualify for authorship, and all those who qualify for authorship are listed.

### CONFLICT OF INTEREST

None declared.

### FUNDING INFORMATION

None.
